# Primary reconstruction of extensive orbital fractures using two-piece patient-specific implants: the Helsinki protocol

**DOI:** 10.1007/s10006-022-01065-y

**Published:** 2022-05-18

**Authors:** Malla I. Salli, Matti Nikunen, Johanna Snäll

**Affiliations:** Department of Oral and Maxillofacial Diseases, Helsinki University Hospital and University of Helsinki, Haartmaninkatu 4E, 00029 HUS Helsinki, Finland

**Keywords:** Orbital fracture, Patient-specific implant, Computer-aided design and manufacturing, CAD-CAM, Diplopia, Globe malposition

## Abstract

**Purpose:**

We present our experience of titanium-milled two-piece patient-specific implants (PSIs) for primary reconstructions of extensive orbital floor and medial wall fractures (EOFMFs) and evaluate their postoperative functional and aesthetic outcomes in relation to commercially available implants.

**Methods:**

We included all patients with primary reconstructions (< 22 days from injury) of EOFMFs treated in our department between January 2011 and October 2020. Extensive orbital floor and medial wall fracture was defined as involvement of orbital floor, medial wall and maxilloethmoidal junction; a fracture defect 5 mm or more; defect size more than a third of both inferior and medial walls; and Jaquiéry classification III or more. Patient characteristics, details of fracture defects and surgeries, postoperative outcomes and implant positions were retrospectively evaluated and compared between study groups.

**Results:**

Nineteen patients were included: 5 with two-piece PSIs and 14 with commercial implants. Implant position was good in 4/5 patients with two-piece PSIs and 2/14 with commercial implants. Revision surgery, globe malposition (GMP) > 2 mm, significant diplopia and poor implant position were more frequent in patients with commercial implants than two-piece PSIs. None of the patients with a good overall implant position had any significant postoperative symptoms.

**Conclusion:**

Extensive orbital fracture reconstructions are somewhat rare, and surgical treatment is associated with a high rate of complications and postoperative symptoms. Titanium-milled two-piece PSIs are well suited for primary reconstructions of EOFMFs, as they lead to more precise reconstructions and fewer postoperative symptoms than commercially available implants.

## Introduction

Orbital wall fractures may be blow-out fractures or fractures that involve the orbital rim in addition to the orbital walls [1]. Orbital fractures usually present with clinical symptoms including swelling and hematoma around the orbit, eye motility disorders, globe malposition (GMP) and diplopia [2]. These findings tend to be more pronounced in cases with extensive orbital wall defects involving both the floor and medial wall (EOFMF) [3], which often require surgical treatment aiming to restore eye function and achieve acceptable cosmesis [4].

Surgical reconstructions of EOFMFs are known to be challenging, especially if the maxilloethmoidal junction is involved as it functions as the anatomical cornerstone of the orbital cavity [5–7]. Although various surgical approaches and methods have been described to treat EOFMFs, persistent postoperative symptoms, including GMP and diplopia, are still relatively common [8]. Precise anatomical restoration of the orbital walls is considered as the main goal of orbital fracture surgery, even though the position of the implant is known not to be the only factor influencing postoperative clinical outcomes [8, 9].

Three-dimensional (3D) technology, including computer-aided design and manufacturing (CAD-CAM) techniques, are increasingly used in the treatment of orbital fractures (10). Orbital implants may be preoperatively prepared by bending them manually on a personalized skull-model or by making a virtual reconstruction by mirroring the unaffected contralateral side on the affected side as a reference [10]. Patient-specific implants (PSI), here defined as implants prepared by the latter method, enable more precise reconstructions than reconstructions with commercial implants [11].

As mentioned, the precision of the bony orbital cavity reconstruction is not the only factor that affects postoperative outcome. In EOFMFs, extensive surgical approaches and substantial surgical handling of the orbital soft tissue may cause marked iatrogenic soft tissue injury. However, if the surgeon attempts to avoid this soft tissue injury, a smaller implant may be chosen and, therefore, a part of the defect may be left uncovered. Both of these situations may lead to residual postoperative symptoms. Thus, two-piece PSIs may help the surgeon to perform a precise anatomical reconstruction, to reduce the iatrogenic orbital soft tissue injury [12] and to decrease the duration of surgery and the requirement for secondary surgery.

The aim of this retrospective study was to present our experience of titanium-milled two-piece PSIs for primary reconstructions of EOFMFs. In addition, we sought to evaluate their postoperative functional and aesthetic outcomes and postoperative complications compared to commercially available implants.

## Materials and methods

### Study design

We sought to compare the outcomes of titanium-milled two-piece PSIs and commercial implants in orbital fracture reconstructions. We retrospectively evaluated all patients with primary orbital fracture reconstruction treated in the Department of Oral and Maxillofacial Diseases, Helsinki University Hospital between January 2011 and October 2020.

### Inclusion criteria and study variables

We included patients, who underwent a primary reconstruction of EOFMF with preoperative and postoperative high-resolution 16-slice computed tomography (CT) imaging into the study. Delay from injury to fracture reconstruction was required to be ≤ 21 days.

Extensive orbital floor and medial wall fracture was defined according to five criteria: orbital floor and medial wall fracture; fracture of maxilloethmoidal junction; ≥ 5 mm fracture defect dislocation, defect size more than a third of both inferior and medial walls; and Jaquiéry classification ≥ III [13].

We collected the following variables and compared them between the study groups: age, sex, delay from injury to surgery, facial fracture type (i.e., isolated orbital fracture without involvement of the orbital rim or impure orbital fractures with involvement of the orbital rim), orbital fracture defect type (i.e., Jaquiéry classification) [13], occurrence of other associated injuries, injury mechanism, surgical approach, duration from injury to surgery and duration of the follow-up period.

Variables regarding clinical postoperative outcomes included the following: requirement of orbital revision surgery; occurrence of GMP > 2 mm, eyelid malposition, any diplopia, diplopia that interfered with daily activities and diplopia that required strabismus evaluation by an ophthalmologist; requirement of additional procedures due to eyelid malposition.

### Evaluation of implant position

The authors evaluated the positions of the implants based on postoperative CT scan images. The overall anterior, medial and posterior positions of the plates were classified as good (i.e. implant resting on sound bony margins, restoring normal contour), acceptable (i.e. implant resting on sound bony margins but not restoring normal contour) and poor (i.e. edge of implant in sinus, not restoring normal contour) as previously described [14]. In addition, we analyzed the defect coverage by the implant (defect fully covered by the implant/maximum defect size left uncovered in mm in any of the CT scan images), location of the defect area left uncovered (medial or lateral to the implant or both) and the resemblance of the implant form to the orbit (good resemblance/implant positioned above the surface of the orbital wall/head of implant pointing into the orbit, or extraocular muscle/implant positioned partly in the ethmoidal or maxillary sinus).

### Virtual planning and manufacturing of patient-specific implants

**Fig. 1:**
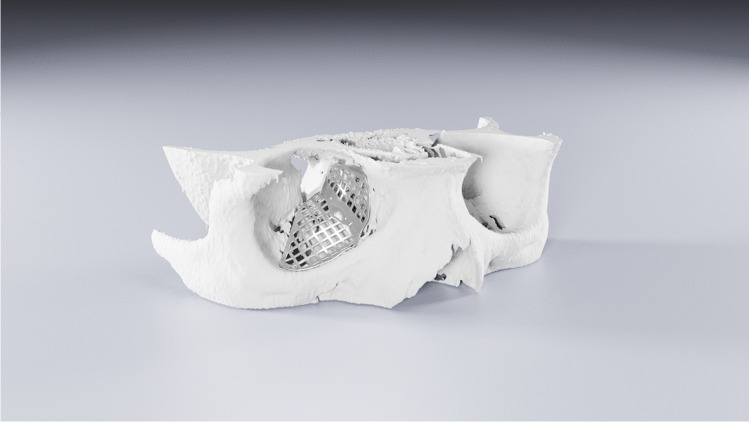
The titanium-milled two-piece implant for right-side orbital fracture was designed virtually. The shape of the orbit was reconstructed by mirroring of the unfractured orbit. The implant consisted of two parts, of which the medial piece was connected to the lateral piece by a groove and conical small hooks

The two-piece PSIs (Fig. 1) were designed preoperatively by one of the authors (J.S.) and engineered using CAD in the Planmeca ProModel™ system (Planmeca Ltd). Mirroring of the unaffected contralateral side was used as a reference for the virtual reconstruction [10]. Two-piece PSIs were designed to rely on at least three intact shelf structures of the orbital structures (anteromedial, anterolateral and posterior): anteriorly on the inner surface of the anterior orbital rim, laterally on the infraorbital groove but not in or over it and medially and posteriorly over the whole fracture defect when possible. The virtually planned design and fit of the implant was tested on a printed 3D model preoperatively to confirm precise fit and to improve the orientation of the surgeon for surgery. The lateral piece of the implant was first placed over the orbital floor. The medial piece was then placed over the fracture defect of the medial wall and connected to the designed groove of the medial edge in floor part implant, where the implants were locked by two or three conical small hooks. The patient-specific implants were computer numerical control (CNC)-milled from titanium (grade 2) alloy blocks to a thickness of 0.3 to 0.4 mm by Planmeca Ltd.

### Statistical analyses

Data were analyzed with IBM SPSS Statistics for Mac (version 24, IBM Corp). The results are shown as means (range) and numbers of named cases (percentages).

## Results

**Fig. 2:**
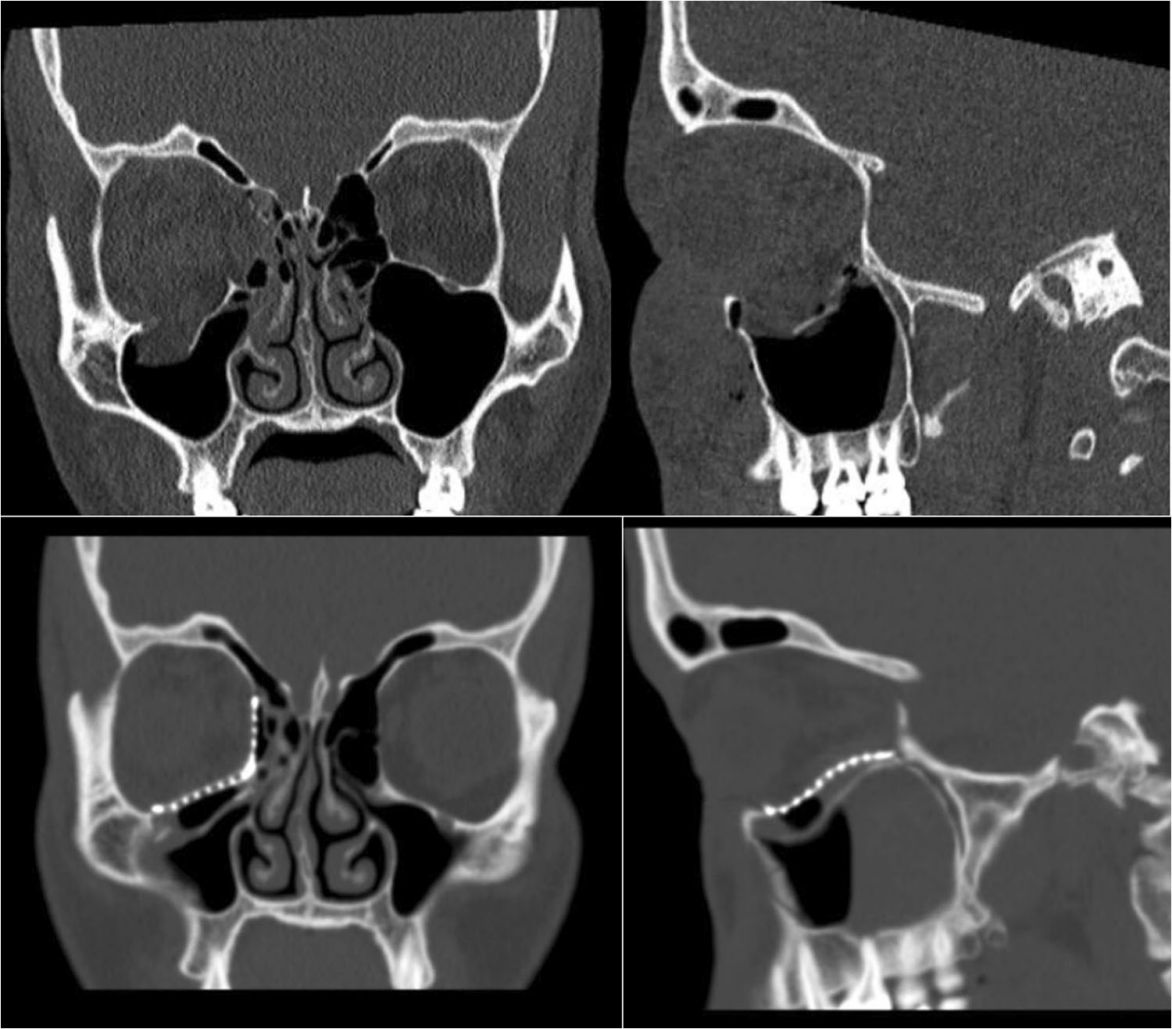
The patient had an extensive right-sided orbital fracture due to assault. The patient’s eye movements were restricted due to soft tissue entrapment on the edges of the fracture. Computer-tomography images showed fracture extension to the posterior orbital third in both the orbital floor and medial wall. The customized two-piece implant was preplanned (Fig. 1), and surgery was conducted 11 days after injury. Postoperative images showed excellent implant fitting

Of the 266 orbital fracture patients requiring primary fracture reconstruction, 19 patients (7%) fulfilled the inclusion criteria and received reconstructions of unilateral EOFMFs (Fig. 2). Five patients had two-piece PSIs and 14 commercial implants (10 patients had prebent orbital implants (MatrixORBITAL, DePuySynthes) and 4 had unprebent orbital meshes (DePuySynthes, MatrixMidface)) (Table 1).

**Table 1:** Patient characteristics and details of trauma and surgery

Variable	PSI (*n* = 5)	Non-PSI (*n* = 14)
Median age, years (range)	42 (24–57)	52 (23–77)
Median delay from injury to surgery, days (range)	11 (4–13)	6 (0–14)
Isolated orbital fracture	2 (40)	8 (57)
Orbital fracture extending to orbital rim(s)	3 (60)	6 (43)
Associated injuries, any	3 (60)	6 (43)
**Intracranial injuries**	1 (20)	5 (36)
**Cervical spine fractures**	0	0
**Cervical vein injury**	0	1 (7)
Treatment delay as days, mean (range)	11 (4–17)	6 (0–14)
Implants used		
Two-piece PSI	5 (100)	0
Prebent implant	0	10 (71)
Unbent mesh	0	4 (29)
Implant fixed with a screw	0	5 (36)
Surgical approach		
Lower eyelid	1 (20)	9 (64)
Transconjunctival	0	3 (21)
Combined transconjunctival and retrocaruncular	4 (80)	2 (14)
Lateral cantholysis associated with the surgical approach	1 (20)	3 (21)

All values are *n* (%) unless otherwise indicated

*PSI* patient-specific implant

Surgical details are presented in Table 1. The delay between diagnosis and primary surgery was slightly greater in patients with a two-piece PSI (mean 11 (4–17) days) compared to other patients (mean 6 (0–14) days). A transconjunctival approach was combined with a retrocaruncural approach in a third of all patients. A lateral cantholysis or canthotomy was performed in a fifth of all patients.

The implants tended to be in a better position in patients with two-piece PSIs (Table 2; Fig. 2) than in patients with commercial implants; none of the two-piece PSIs were positioned poorly. Most of the suboptimal and poor positionings of the commercial implants were located in the posterior and medial parts of the fracture defect (Table 2). Moreover, only one patient with a two-piece PSI (20%) had 5 mm or more of the fracture defect left uncovered; the respective number was 93% in patients with commercial implants (Table 2).

**Table 2:** Positions of the implants on the postoperative CT scan

Variable	Two-piece PSI (*n* = 5)	Non-PSI (*n* = 14)
Overall		
Good	4 (80)	2 (14)
Suboptimal	1 (20)	10 (71)
Poor	0	2 (24)
Anteriorly		
Good	5 (100)	14 (100)
Medially		
Good	5 (100)	8 (57)
Suboptimal	0	4 (29)
Poor	0	2 (14)
Posteriorly		
Good	4 (80)	7 (50)
Suboptimal	1 (20)	6 (43)
Poor	0	1 (7)
Implant covering the fracture defect		
Defect fully covered by the implant	2 (40)	0
≥ 5 mm of the defect left uncovered	1 (20)	13 (93)
1–4 mm of the defect left uncovered	2 (40)	2 (14)
5–10 mm of the defect left uncovered	1 (20)	8 (57)
> 10 mm of the defect left uncovered	0	5 (36)
Location of the uncovered fracture defect		
Medial to the implant	3 (60)	10 (71)
Lateral to the implant	0	0

All values are *n* (%) unless otherwise indicated

*PSI* patient-specific implant

Postoperative long-term follow-up data were available from 14 patients (Table 3). Overall, at the final follow-up, eight patients (57%) experienced any diplopia, which disturbed daily activities in two patients (14%) and required prism glasses in one patient (7%). Moreover, of patients with commercial implants, five (50%) had any lower lid malposition, two (14%) had significant globe malposition, and two (14%) required revision surgery. Postoperative clinical complications were more frequent in patients with commercial implants than two-piece PSIs (Table 3). Patients with two-piece PSIs did not require revision surgery or suffer from GMP > 2 mm (Table 3).

**Table 3:** Clinical postoperative outcomes of patients with extensive orbital fracture reconstruction

Variable	two-piece PSI (*n* = 4)	Non-PSI (*n* = 10)
Follow ups actualized as planned	4/5 (80)	10/14 (71)
Length of the follow-up period among the patients who participated in the follow-ups, days (range)	99 (43–160)	273 (24–804)
Postoperative complications		
Revision surgery	0	2 (14)
Significant globe malposition (> 2 mm)	0	2 (14)
Any lower lid malposition	0	5 (50)
Lower lid malposition requiring surgical procedures	0	2 (20)
Any diplopia, *n* (%)	2 (50)	6 (60)
Diplopia disturbing daily activities	0	2 (20)
Diplopia requiring evaluation at the strabismus policlinic	1 (20)	1 (10)
Diplopia requiring strabismus surgery	0	0
Strabismus requiring prism glasses	0	1 (10)

All values are *n* (%) unless otherwise indicated

*PSI* patient-specific implant

Patients with a poor implant position tended to have significant postoperative clinical symptoms; all of these patients had both significant GMP and some degree of diplopia (Table 4).

**Table 4:** Implant position vs postoperative clinical symptoms

Variable	Significant globe malposition (*n* = 2)	Any diplopia (*n* = 8)	Diplopia disturbing daily activities (*n* = 2)
Overall			
Good (*n* = 5)	0	2 (40)	0
Suboptimal (*n* = 7)	0	4 (57)	1 (14)
Poor (*n* = 2)	2 (100)	2 (100)	1 (50)
Medially			
Good (*n* = 10)	0	6 (60)	1 (10)
Suboptimal (*n* = 2)	0	0	0
Poor (*n* = 2)	2 (100)	2 (100)	1 (50)
Posteriorly			
Good (*n* = 8)	1 (13)	3 (38)	0
Suboptimal (*n* = 5)	0	4 (80)	1 (20)
Poor (*n* = 1)	1 (100)	1 (100)	1 (100)

All values are *n* (% of the total number of patients in each category) unless otherwise indicated

## Discussion

In this single-centre retrospective study, we presented a series of cases with reconstructions of EOFMFs. We found excellent postoperative outcomes in patients with two-piece PSIs; they had anatomically more precise reconstructions with less postoperative symptoms compared to patients with commercial implants despite their larger orbital defects. As EOFMFs are rare and may be challenging to reconstruct, our results suggest that two-piece PSIs can be recommended for their primary reconstructions to avoid further revision surgeries and other suboptimal outcomes.

The aims of orbital reconstructions are to restore the anatomy of the orbital cavity [9, 15], retrieve orbital soft tissue content, eliminate unstable bony fragments and identify stable bone platforms while causing minimal iatrogenic injury [3, 16]. This is essential to achieve acceptable postoperative outcomes and to avoid complications [3, 16, 17]. As verified by our results, most of the technical challenges related to reconstructions of EOFMFs are located in the medial and posterior walls and in the inferomedial strut of the orbital cavity, as recognizing these structures post-traumatically is difficult. Moreover, dissection of these structures must be carefully performed due to the proximity of several important structures, including the medial palpebral ligament, lacrimal system, ethmoidal arteries and optic nerve [3].

In our series, most of the patients with commercial implants had malpositioned implants. In contrast, this was observed in only one patient with a two-piece PSI and was not clinically relevant. These findings reflect the challenges related to the process of fitting and aligning commercial implants, which may be time consuming and operator dependent. Importantly, these results also emphasize the advantages of CAD-CAM technology in reconstructing EOFMFs, as personalized CAD-CAM implants lead to more precise orbital volumetric reconstruction compared with traditional reconstruction methods [18, 19]. Additionally, CAD-CAM implants can be modelled preoperatively, which may further decrease the possibility of errors in implant positioning and reduce surgery duration [11].

Enophthalmos, restricted extraocular muscle motility and diplopia are a typical postoperative complication related to extensive orbital wall fractures [3, 9, 20, 21]. Postoperative symptoms may be caused not only by the fracture defect and changes in the volume of the orbital cavity [3], but also by orbital soft tissue injury and herniation via compromised soft tissue function and eye movements [20, 22, 23]. Some previous studies [8, 23, 24] revealed that the development of unfavourable clinical outcomes was not associated with any of the radiological predictors regarding the fracture or the implant type or position, which our results partly support; postoperative clinical symptoms were not only related to implant position. However, poor implant position tended to lead to more pronounced postoperative symptoms when compared with good or suboptimal position. This indicates that anatomically precise reconstruction is still a relevant goal for orbital fracture surgery, for which 3D technology is a beneficial tool.

The delay between fracture diagnosis and primary surgery was longer in patients with PSIs than commercial implants. However, this difference was not clinically significant, as orbital fracture surgery can be safely performed within the first weeks of the trauma without leading to inferior postoperative outcomes [25].

There is debate that the transconjunctival-retrocaruncular approach may cause postoperative complications, including persistent inferior oblique malfunction, inferior canalicular obstruction and scarring, which may result in diplopia [26]. In our series, no signs of complications related to the surgical approach were found, which is consistent with some previous studies [7, 27]. On the contrast, transconjunctival-retrocaruncular approach provides good visibility to the narrow operative field with potential avoidance of excessive tissue stretching, lateral canthotomy or cantholysis or skin incisions during the surgery when used with two-piece PSIs.

In our centre, the Helsinki protocol of two-piece PSIs is fairly simple and quick; the implant can be acquired within 1 day of order and thus does not cause a significant delay in surgery. Even if the manufacturing costs of PSIs for primary orbital reconstructions are evidently higher than using commercial implants, they lead to more precise implant positioning and better clinical outcomes. Thus, the costs of the overall treatment may eventually decrease and lead to less morbidity and stress to the patient. However, due to the importance of cost-effectiveness, the performance of primary orbital reconstruction with PSIs could be started in patients with large fractures, as they generally have poorer postoperative outcomes than patients with single-wall fractures.

The correct placement of PSIs was easily evaluated without intraoperative navigation by the good fitting of the PSI over the fracture defect and the proper fixation between the two parts of the implant. Thus, two-piece PSIs may at least partially replace intraoperative navigation and imaging, which were not available in the present study. Despite the screwless fixation of two-piece PSIs, no failures of the attachment between the implant parts occurred.

The greatest limitation of this study was the low number of patients, which precludes definite conclusions. Additionally, the retrospective and non-comparative study design and the limited rate of actualized clinical follow ups were also weaknesses. Moreover, due to the low number of patients and the variety of procedures performed during the primary surgery of the fracture reconstruction, this study did not assess the effect of PSIs on surgery duration.

## Conclusions

Titanium-milled two-piece PSIs are well suited for primary reconstructions of EOFMFs. These patients had fewer postoperative clinical symptoms than patients with commercial implants. This was possibly due to the less invasive surgical approaches, reduced iatrogenic soft tissue injury and greater precision of reconstruction. Therefore, we recommend the described two-piece PSI method for these most challenging primary orbital fracture reconstructions. However, to achieve even better postoperative outcomes for all orbital fracture patients, future research should focus on the role of damage to intraorbital soft tissues [23, 28] and the importance of atraumatic surgical techniques in addition to the precision of the reconstruction.
